# Open Tracheostomy for Critically Ill Patients with COVID-19

**DOI:** 10.1155/2020/8861013

**Published:** 2020-11-30

**Authors:** Estefanía Hernández-García, Mar Martínez-RuizCoello, Andrés Navarro Mediano, Nuria Pérez-Martín, Victoria García-Peces, Carlos Velayos, Belen Rodríguez-Campoo, Guillermo Plaza

**Affiliations:** ^1^Department of Otorhinolaryngology, Hospital Universitario de Fuenlabrada, Universidad Rey Juan Carlos, Madrid, Spain; ^2^Department of Intensive Care, Hospital Universitario de Fuenlabrada, Universidad Rey Juan Carlos, Madrid, Spain; ^3^Department of Anesthesiology, Hospital Universitario de Fuenlabrada, Universidad Rey Juan Carlos, Madrid, Spain

## Abstract

**Background:**

COVID-19 is a worldwide pandemic, with many patients requiring prolonged mechanical ventilation. Tracheostomy can shorten ICU length of stay and help weaning. *Aims/Objectives*. To describe the long-term evolution of the critically patient with COVID-19 and the need for invasive mechanical ventilation and orotracheal intubation (OTI), with or without tracheostomy. *Material and Methods*. A prospective study was performed including all patients admitted to the ICU due to COVID-19 from 10^th^ March to 30^th^ April 2020. Epidemiological data, performing a tracheostomy or not, mean time of invasive mechanical ventilation until tracheotomy, mean time from tracheotomy to weaning, and final outcome after one month of minimum follow-up were recorded. The Otolaryngology team was tested for COVID-19 before and after the procedures.

**Results:**

Out of a total of 1612 hospital admissions for COVID-19, only 5.8% (93 patients) required ICU admission and IOT. Twenty-seven patients (29%) underwent a tracheostomy. After three months, within the group of tracheotomized patients, 29.6% died and 48.15% were extubated in a mean time of 28.53 days. In the nontracheostomized patients, the mortality was 42.4%.

**Conclusions:**

Tracheostomy is a safe procedure for COVID-19 and helps weaning of prolonged OTI. Mortality after tracheostomy was less common than in nontracheostomized patients.

## 1. Introduction

COVID-19 is a worldwide pandemic that is putting unprecedented demand on intensive care units (ICU) and hospitals [1]. Therefore, the number of orotracheal intubations (OTI) has increased to the same extent, requiring in most cases prolonged invasive mechanical ventilation.

There is current uncertainty about the role of tracheostomy for weaning of mechanically ventilated patients with COVID-19. Several recommendations, guidelines, and consensus have been published recently on the indication and precautions of such airway procedure during COVID-19 pandemic [2–6]. Tracheostomy is an intervention performed very frequently by otorhinolaryngologists around the world, ensuring the airway quickly, with very clear and reproducible steps. These surgical steps have been adapted by several authors to improve results and avoid complications during COVID-19 pandemic [7–9].

Nevertheless, the role of tracheotomy in critically ill patients with COVID-19 is yet unknown, and there is no clear indication of the timing of tracheotomy after OTI in these patients [2, 5]. Indeed, tracheostomy offers several other advantages such as reducing sedative and paralytic medical support, facilitating airway suctioning and clearance of secretions, and preventing tracheal stenosis. However, due to a number of factors including prognosis, optimal utilization of healthcare resources, and the safety of healthcare workers when performing such a high-risk aerosol generation procedure, several authors do not approve it when COVID-19 might be contagious [2, 9–12].

The objective is to describe the evolution of the critically ill patient with COVID-19 infection and the need for invasive mechanical ventilation and tracheostomy, mean time to weaning, mean length of stay in the ICU, possible complications, infection rate of the surgeons, and outcome.

## 2. Material and Methods

This prospective study was approved by our local Board of Ethics and included all consecutive patients admitted to the ICU of a tertiary care teaching hospital who required invasive mechanical ventilation and OTI from 10^th^ March 2020 to 30^th^ April 2020 and had a minimum follow-up of two months. We included all patients regardless of age admitted to the ICU with a diagnosis of COVID-19. Patients admitted to the ICU for other reasons were excluded.

If the patient met weaning targets such as FiO_2_ less than 40%, PEEP < 8, PaO_2_/Fi_O_2 > 200, and pressure support < 8 cm H_2_O, extubation could be reached between 7 and 14 days, and tracheostomy should be postponed. If patients were far from reaching weaning targets, tracheostomy was proposed.

When indicated, regardless of RT-PCR positivity of the patients, tracheostomies were performed by a reduced number of providers limited to the strictly essential members. Tracheostomies were arranged in groups of 2 or 3 per session to minimize personal protective equipment usage. Healthcare workers wore water-resistant gowns, caps, boots, double gloves, goggles, and FFP3 masks providing protection against droplet-based transmission, with full-face transparent shields on top.

Open surgical tracheostomy was performed by two experienced otolaryngologists following published guidelines [3, 12]. They were done bedside in the ICU, avoiding transportation to the OR. A horizontal skin incision was made; the thyroid isthmus was sectioned if needed. Suctioning and cautery were minimized after preoxygenation (oxygen 100% for 2-3 minutes); mechanical ventilation was ceased during tracheal incision to minimize viral aerosolization, paying particular care not to pierce the inflated cuff. To further reduce aerosolization, complete paralysis was obtained to avoid coughing. The surgeon incised the trachea between rings II-III. Under direct view from the tracheotomy, a cuffed nonfenestrated tracheostomy tube was placed. Stethoscope auscultation was avoided, and confirmation of correct tracheal tube placement relied on end-tidal gas sampling and chest movements. During tracheostomy, the trachea was effectively sutured to the skin at the end of the procedure, when two trachea stitches are given to the skin to ensure the airway. Later, when the tracheostomy was closed, these stitches were removed. A doffing procedure was performed by team members individually and one at a time, following a standardized sequence to avoid self-contamination and under supervision of a dedicated inspector.

We analyzed the epidemiological data, previous comorbidities, initial symptoms, and mean time of symptoms to consult in the emergency department. Also, we recapitulate the result of the diagnostic RT-PCR test, number of tests required until diagnosis, mean time until admission to the ICU and OTI or presence of bilateral or unilateral pneumonia, if it was necessary to perform a tracheostomy or not, mean time of invasive mechanical ventilation until tracheotomy, mean time from tracheotomy to weaning, mean time of ICU stay, treatment received, main recorded systemic complications, and final outcome (discharge or death). We also collected serology and PCR from the surgeons to assess the possibility of contagion after the procedure.

## 3. Results

We present a prospective cohort of 1612 patients admitted for COVID-19 from 10^th^ March 2020 to 30^th^ April 2020 at a university hospital, representing a 0.8% of the total population of the city belonging to this health area.

Of these patients, ninety three (5.8%) were admitted to the ICU with necessity of OTI. Of those 93 patients, 68.8% were males, and the mean age was 62.8 years (range 36–77) (Table 1). The most frequent comorbidity was obesity (51.62%), followed by hypertension (49.5%), dyslipidemia (30%), diabetes (30%), and smoking (28%).

COVID-19 was diagnosed after a mean of 2.1 number of RT-PCR per patient to achieve a positive result, although two patients died during follow-up due to severe bilateral pneumonia and clinical diagnosis of COVID-19 without a positive RT-PCR obtained.

After a follow-up time of three months from admission to the ICU, out of 93 patients, 49 (52.6%) were discharged within 30 days, 8 (8.6%) were discharged after a prolonged ICU stay, and 36 (38.7%) died. Excluding deaths, the mean ICU stay was 21.58 days (range 2–54).

Twenty-seven patients (29%, 27/93) underwent a tracheostomy. The mean time from OTI to tracheostomy was 17 days (range 8–32). After ventilatory improvement, 48.15% (13/27) were extubated and the mean time from tracheostomy to weaning was 28.53 days (range 13–54). The mean ICU stay in the tracheostomy patients, excluding deaths, was 31.92 days (range 18–54).

Within the group of tracheotomized patients, 29.6% (8/27) died, although no procedure-related mortality was observed. Six patients suffered a long ICU stay after tracheostomy to reach weaning from mechanical ventilation through the tracheal cannula, with a mean stay in ICU of 40.25 days (range 27–53).

In the nontracheostomized patients, the mortality rate was 42.4% (28/66), while 36 patients (54.5%) were extubated after a mean time of 11.34 days (range 1–28). In these patients, the mean ICU stay, excluding deaths, was 13.48 days (range 2–31). These long-term data differ from early results within the first two weeks (Table 2).

Regarding complications after tracheostomy, three cases (11.1%) required revision due to sustained bleeding, thirteen (48.2%) cases had minor ventilation problems, such as cannula leak, and only one case needed treatment for pneumothorax. During follow-up, tracheostomies were closed by pressure vending avoiding the use of contaminating fenestrated cuffs, without major complications.

Out of six surgeons and three nurses involved, none of them acquired COVID-19 after 27 tracheostomies performed, as shown by negative RT-PCR and serologies.

## 4. Discussion

COVID-19 pandemic has been a great challenge for all health systems in the world. Many hospitals have been overwhelmed and have needed to expand their ICUs exponentially as intubation points and new ventilators were required, requesting the help of many professionals beyond the intensivists.

According to published literature, COVID-19 mortality is around 2–5%, and there are few publications on the percentage of those infected requiring admission to an ICU [1]. In our study, 5.8% of COVID-19 patients required ICU admission, requiring OTI, considering tracheostomy as another aid to these patients.

The indication of tracheotomy in this period is controversial. There are several authors who consider that it should be postponed as much as possible, due to the high risk of contagion to healthcare workers and its apparently few benefits [2, 3, 10, 13]. However, other authors promote its realization improving the management of patient comfort, reducing the amount of sedatives or medication required, or reducing the pulmonary dead space [4, 5, 14–20]. Currently, this decision is made jointly with intensive care professionals, anesthetists, and otorhinolaryngologists.

In our series, the mean time from OTI to open tracheostomy was 17 days (range 8–32). We followed a protocol advising for late tracheostomy, following most guidelines [5, 6], and similar to other studies, such as study by Turri-Zanoni et al. [16], reporting on 32 tracheostomies after a mean intubation period of 15 days, or Riestra-Ayora et al. [17] on 27 open tracheostomies, with a mean time from OTI to tracheostomy of 13 days.

However, most authors have reported their early results after tracheostomy in COVID-19, only 2–4 weeks after surgery was done [14–19]. In the present study, we have observed how tracheostomy helps weaning and cure in almost half the cases, allowing an overall mortality after ICU stay to be less than 45% in severe COVD-19 patients (Table 2). However, tracheostomy results in longer stay at the UCI and increases care from healthcare workers to achieve weaning and decannulation.

Regarding the protection against COVID-19, there are many articles alarming off an increased exposure of otorhinolaryngologists, since most of the explorations and surgeries we carry out have a high generation of aerosols [12]. In our study, we have observed no otorhinolaryngologists have become infected while performing tracheotomies, as in other reports.

As limitations of this study, it should be considered that our sample is small (*n* = 27) but rapidly accumulated in an overpassed ICU. Moreover, COVID-19 patients usually stay very long in ICU and even more in patients with tracheostomy and several comorbidities from COVID-19. These factors seem to make weaning more difficult. More long-term studies are necessary to observe complications and to evaluate which predictors are more relevant.

In conclusion, the majority of patients with severe COVID-19 infection were males in their 60s and with cardiovascular comorbidities and obesity. When OTI is prolonged, it is recommended to perform a tracheostomy to avoid complications, the exact moment should be individualized. COVID-19 infection involves a long ICU stay in most patients, requiring invasive prolonged mechanical ventilation. Tracheostomy is a safe procedure for COVID-19 and helps weaning of prolonged OTI. The mortality of patients admitted to the ICU is high, being somewhat less in the group that had a tracheostomy done. With protection, tracheostomy is a safe procedure.

## Figures and Tables

**Table 1:** Clinical characteristics of COVID-19 tracheotomies.

Male/female (tracheostomy group)	63 (19)/30 (8)
Age	62.8 years (range 36–77)

Comorbidities (tracheostomy group)	Hypertension 49.5% (74%)
	Dyslipidaemia 30%
	Diabetes 30% (33%)
	Smoking 28% (ex 19%, active 30%)
	Obesity 51.6% (52%)

Initial symptoms	Fever 74.1%
	Cough 63.4%
	Dyspnoea 52.7%

BMI kg/cm^2^ (tracheostomy group)	31.68 (30.49)

**Table 2:** Comparison between early and long-term results and tracheostomy vs nontracheostomy in COVID-19 patients.

ICU stay in days (total group)	Early findings		>30 days follow-up	
	17.4		20.29	
	Tracheostomy group	Nontracheostomy group	Tracheostomy group	Nontracheostomy group

Cease of mechanic ventilation (%)	37.03%	46.9%	48.15%	54.5%

Time until death	8.9 days	11.6 days	18.53 days	11.34 days

Mortality	29.6%	36.4%	29.6%	42.4%

## Data Availability

Study data are available in the link https://www.dropbox.com/s/0p0mxpwiq9685ag/COVID%20estudio%20INTUBAC-TRAQ%20vANONIMA.xlsx?dl = 0REFERENCES.
